# Exploring the Experiences of Women Who Develop Restrictive Eating Behaviours After Bariatric Surgery

**DOI:** 10.1007/s11695-020-04424-4

**Published:** 2020-02-14

**Authors:** Charlotte Watson, Afsane Riazi, Denise Ratcliffe

**Affiliations:** 1 Department of Psychology, Royal Holloway, University of London, London, UK; 2 Bariatric Surgery Psychology Department, Chelsea and Westminster NHS Foundation Trust, Fulham, London, UK

**Keywords:** Bariatric medicine, Bariatric surgery, Eating disorders, Anorexia nervosa, Obesity, Body image, Weight stigma, Post-surgery eating disorders, Eating pathology

## Abstract

**Objective:**

This study aimed to explore the experiences of women who had developed excessively restrictive eating behaviours following bariatric surgery.

**Method:**

Five female participants, who were at least nine months post-bariatric surgery and exhibiting restrictive eating behaviours, were recruited from Bariatric Surgery Psychology Services and asked to complete qualitative face-to-face semi-structured interviews. The data was analysed using interpretative phenomenological analysis (IPA).

**Results:**

Three super-ordinate themes emerged: (1) experiences of weight stigma and weight history on self, (2) the impact of loose skin, (3) thoughts about food and disordered eating patterns. These captured the impact of past weight-related experiences—including weight stigma, intense fears of weight gain, negative cognitions about the self, the impact of excess skin, changes in the way the women thought about food and restrictive eating behaviours.

**Discussion:**

This study is one of the first to specifically explore restrictive eating disorders after bariatric surgery using a qualitative approach. The findings of this study may offer helpful aspects for professionals to hold in mind when identifying individuals with problematic restrictive eating behaviours following bariatric surgery. Body contouring surgery, internalised weight bias and weight stigma are explored in relation to the post-bariatric surgery treatment pathway. The current diagnostic criterion for anorexia nervosa are discussed to highlight difficulties in diagnosing this presentation in the post-bariatric surgery population, where people can have BMIs over 25 kg/m^2^ but are severely restricting energy intake.

## Introduction

Bariatric surgery is regarded as the most effective treatment for severe obesity [1]. Bariatric surgery procedures are not without risk and research attention is being given to post-surgery maladaptive eating patterns due to their influence on outcomes [2, 3]. However, there has been limited focus on the increasing number of people who are developing more restrictive eating disorder patterns. Increasingly, clinicians are seeing post-surgery clients who are starved, defined by evidence of malnutrition, but have a BMI over 17.5 kg/m^2^ and 18.5 kg/m^2^ [4, 5], which historically have been clinical indicators of a person being underweight or having anorexia nervosa (AN) [6, 7].

The restrictive eating requirements recommended after bariatric surgery are strongly associated with extreme weight loss [5]. Portion sizes are significantly reduced, and people are often encouraged to weigh their food and strictly control their calorie intake. Certain food types (for example very sweet foods) and certain eating habits (for example eating too fast or not chewing food enough) are discouraged as they can cause pain and self-induced or spontaneous vomiting. These behaviours around food are part of an individual’s normal adjustment and adherence to post-surgery life and are often encouraged by professionals; however, food avoidance and restriction are also commonly seen in AN [4, 8, 9]. Changes experienced following surgery (i.e. rapid weight-loss and dietary restraint) may trigger or lead to the development of, restrictive “AN-like” eating pathology, characterised by greater than usual weight loss after surgery, fear of weight regain, dietary restriction and disturbances in self-perception of shape and weight [9, 10]. Due to the under-researched nature of this area, the prevalence of restrictive eating disordered behaviour after bariatric surgery is unknown, though it is becoming more common [4, 7].

There has been a debate in the literature around whether a specific diagnosis is needed to differentiate between development of unhealthy behaviours and cognitions post-bariatric surgery (e.g. Post-Surgical Eating Avoidance Disorder, PSEAD [11]) and the specialised management of a person’s nutritional needs due to gastrointestinal system alterations [4, 8, 9, 11, 12]. Segal, Kussunoki and Larino [11] highlighted that clinicians were seeing post-surgical clients with a marked and sustained change in their relationship with food associated with an intense fear of regaining weight and food restriction, but due to “normal” BMIs were not fulfilling the full DSM-IV criteria for AN [6], despite relevant signs of malnutrition. They proposed that although other diagnostic classifications (e.g. Eating Disorder Not Otherwise Specified, EDNOS) could have been used, they may not have been sufficient to comprehensively describe this specific population due to the eating behaviours that may be specific to post-bariatric surgery clients. This increase in presentation contributed to the removal of the low body weight requirement in the DSM-V criteria for AN, and a broader term looking at the restriction of energy intake was introduced [7, 13–15].

Although DSM-V has now removed the low-body weight requirement for AN, establishing what is considered a normal or ideal BMI after bariatric surgery and what should be considered as underweight has contributed to challenges in identifying this specific presentation. Often, a BMI of 25 kg/m^2^ is used as a target weight [16]; however, it has been debated that for people who have been severely obese, getting to this BMI is extremely unlikely and usually a cause for concern because of being accompanied by evidence of malnutrition, even though it is a BMI that is considered normal weight [4, 17, 18].

Assessing weight and shape concern is complicated after bariatric surgery. Fear of weight (re)gain is probably quite realistic in this population; the majority of people seeking bariatric surgery have a yo-yo pattern of dieting and weight loss, characterised by multiple experiences of losing, or trying to lose, large amounts of weight and then regaining it [10, 19, 20]. In addition, weight (re)gain for someone who has had bariatric surgery is likely to trigger concerns about returning to a marginalised group and pervasive and intense weight-related stigmatization [21, 22]. Research has shown that individuals of average body weight are treated more favourably than those who have excess body weight [23, 24]. This is likely to differ from the weight gain concerns of the majority of people who gain weight following AN, who would have been unlikely to reach BMIs of greater than 35 kg/m^2^ [7]. Following bariatric surgery, weight is strictly monitored by professionals and is used as a key outcome measure. Given the individual’s weight history and the clinical service context, it is understandable that individuals may become preoccupied with weight loss [25, 26].

Shape concern will differ due to the common presence of hanging, loose, overstretched skin, skin envelopes and fat deposits due to the rapid weight loss that follows bariatric surgery [27]. Over 70% of bariatric surgery clients will develop excess skin [28]. Development of excess skin can cause disappointment with the outcome of bariatric surgery, increase body image dissatisfaction, and motivate people to seek body contouring surgery [22, 29, 30]. Understanding around why excess skin is more of an issue for some people and not others remains unclear. Possible factors include age, how much the skin has been stretched by a person’s pre-operative weight, unique changes in the skin following surgery, as well as the amount and speed of weight loss post-surgery [28, 31]. Excess skin is commonly located in the abdomen, upper arms and thighs and is associated with negatively impacted quality of life, as well as multiple complications such as fungal infections; abdominal and back pain; problems with personal hygiene, movement and urination; and difficulties in physical activity, dressing and sexual functioning [32–34].

To date, the limited research looking at the development of restrictive eating behaviours after bariatric surgery has consisted of case studies and reports [e.g. 4, 5, 9, 25, 35–37]. While useful in giving a context to the area, they cannot provide more in-depth insight into people’s personal experiences. This study aimed to explore the lived experiences of women who develop restrictive eating disordered behaviours following bariatric surgery. The objectives were to identify the main experiences described by individuals in order to aid identification of clients who may be at risk of developing restrictive eating behaviours after bariatric surgery.

## Method

The study used a qualitative design with individual face-to-face, semi-structured interviews.

### Sample

Interpretative phenomenological analysis (IPA) was the chosen analytic strategy. A sample size of between four to ten interviews is recommended to credibly show patterns across the data but also keep the focus on the unique individual experiences [38–40]. Five women who had undergone bariatric surgery were interviewed. Table 1 summarises their general demographics.

**Table 1:** Summary of the general demographics for the five participants

Participant	PA01	PA02	PA03	PA04	PA05
Age	31	29	35	38	55
Ethnicity	White—Irish	White—English	Any other Asian background	White—English	White—English
Relationship status	Engaged	Engaged	Married	Married	Married
Employment status	Full-time employment	Not employed	Part-time employment	On maternity leave	Part-time employment
Type of surgery	Roux-en-Y Gastric bypass	Roux-en-Y Gastric bypass	Roux-en-Y Gastric bypass	Roux-en-Y Gastric bypass	Roux-en-Y Gastric bypass
Time since surgery (Date of surgery)	8 years (30.10.08)	1 year (16.12.15)	9–10 months (24.02.16)	3 years (25.09.13)	2 years (16.12.14)
Height (m)	1.64	1.73	1.49	1.75	1.57
Weight (kg) and BMI (kg/m^2^) before surgery	143.60 kg 53.38	140.90 kg 47.07	103.00 kg 46.39	161.00 kg 52.56	104.00 kg 42.18
Current weight (kg) and BMI (kg/m^2^)	94.00 kg 34.94	88.90 kg 29.70	64.50 kg 29.05	97.00 kg 31.67 (Recently gave birth)	76.00 kg 30.83
Lowest weight (kg) BMI (kg/m^2^)	82.60 kg 31.71	88.90 kg 29.70	64.50 kg 29.05	77.50 kg 25.30	75.20 kg 30.50
% of Total weight loss (%TWL)	42.48	36.91	37.38	51.86	27.69

Only female participants were recruited due to the prevalence of eating disorders being higher in females with a 10:1 female-to-male ratio of AN in clinical populations [14]. In addition, 74% of people seeking bariatric surgery are women [41].

Eligible participants were a minimum of nine months post-surgery in order to allow enough time to go through the texture progression of post-surgery diet and establish a pattern of eating. No upper limit on years post-surgery was set. Previous studies demonstrated that people could present to services with disordered eating behaviours even 26 years after surgery [4]. The average time elapsed since surgery in this sample was 35.60 months (SD = 35.34 months), which equates to 2.90 years, and ranged from 9/10 months up to 8 years.

All participants met the DSM-V criterion for AN [14] (see Table 2) apart from low body weight. The average percent of total weight loss (%TWL) for the sample was 39.26% (SD = 8.83%; range 27.69–51.86%), and the average lowest BMI for the sample was 29.25 kg/m^2^ (SD = 2.42 kg/m^2^; range 25.30 kg/m^2^–31.71 kg/m^2^) [16, 42].

**Table 2:** DSM-V [14] criterion for anorexia nervosa

Criterion	Description
A	Restriction of energy intake relative to requirements, leading to a significantly low body weight in the context of age, sex, developmental trajectory, and physical health. Significantly low weight is defined as a weight that is less than minimally normal or, for children and adolescents, less than that minimally expected.
B	Intense fear of gaining weight or of becoming fat or persistent behaviour that interferes with weight gain, even though at a significantly low weight.
C	Disturbance in the way in which one’s body weight or shape is experienced, undue influence of body weight or shape on self-evaluation, or persistent lack of recognition of the seriousness of the current low body weight.

### Procedure

Over approximately four months, bariatric psychologists identified potential participants who met the inclusion criteria and invited them to take part during routine clinic appointments. Five women were identified and approached and all five women decided to take part. The interviews took place either at a hospital clinic or in the participant’s home. The interviews were audio recorded and transcribed and lasted between 41 and 128 min.

### Data Analysis

The data was analysed using IPA as described in Smith et al. [38], and general guidelines for assessing quality and validity in qualitative research were consulted [43–45]. The IPA method explores people’s lived experiences and the meaning they attach to these experiences. IPA has theoretical roots in phenomenology, hermeneutics and idiography. IPA views humans as sense-making creatures, and therefore, the accounts that people provide will reflect their attempts to make sense of their own experience. IPA focuses on the particular through detailed analysis and is committed to understanding how particular experiential phenomena have been understood from the perspective of particular people, in a particular context (for further information refer to Smith, Flowers, & Larkin 2009, Chapter Two) [38].

Understanding the development of restrictive eating disordered behaviour after bariatric surgery is still in the early stages. Examining people’s lived experience is a natural next step because it helps to identify how people may construct meaning of their behaviours and “gives voice” to the people experiencing this phenomenon.

The process of data analysis in IPA starts with analysing the first case’s interview transcript in detail before moving on to the next. The steps involved are “Reading and Re-reading” so that the researcher can immerse themselves in the data; “Initial Noting” of exploratory descriptive, linguistic and conceptual comments; “Developing Emergent Themes” from the exploratory comments to produce a theme that aims to capture and reflect an understanding of what is crucial at that point; and “Searching for Connections” looking at how the emergent themes fit together to create sub-themes. Once all the cases have been analysed all the sub-themes are compared to find patterns or super-ordinate themes. In this study, IPA revealed three super-ordinate themes, which are summarised by their sub-ordinate themes, and were represented by the majority of participants’ accounts (see Table 3).

**Table 3:** Master table of themes

Super-ordinate theme	Sub-ordinate theme	Supporting quotes
1. Experiences of weight stigma and weight history on self	1.1 Influence of past experiences related to weight on the present 1.2 Fear of putting on weight and going back to before 1.3 Struggling with my mind—internal battles	“I can vividly remember how I felt before and what people would used to say to me walking down the street and what people would shout out to you I can physically still remember how that made me feel” (Participant 4) “… the reason why I struggle with the food is I do not want to be here again I’m scared stiff of putting that weight back on” (Participant 5) “… anything to do with my weight, body image, food all fall into the ‘this is why you are crap’ category without fail” (Participant 1)
2. The impact of loose skin	2.1 It reminds me of what I was before—excess skin as a reminder 2.2 You cannot tell I’ve lost weight because of it—excess skin hides weight loss 2.3 I look like melted candle woman—excess skin is unsightly	“… it’s always there a reminder of what it was before what I was like before” (Participant 3) “I’ve got all of the excess skin and it’s just you cannot I cannot tell I’ve lost weight because of it” (Participant 2) ” I look like melted candle woman, or a balloon that’s been popped and it’s all wrinkly and stuff with all the excess skin” (Participant 1)
3. Thoughts about food and disordered eating patterns	3.1 The way I feel about food 3.2 Disordered eating behaviours	“… if I go anywhere that I’m not used to or it’s tapas or anything that you get a lot of food put in front of you I panic […] just looking at it panics me” (Participant 4) “… there’s just no logic when it comes to food there is just no logic […]the logic is screwed […] when it comes to food there is no logic” (Participant 1) “I was skipping meals then it was see if I can go all day without anything to eat you know and then perhaps eat something at night […] if I can go as long as possible without eating I do” (Participant 5)

## Results

### Experiences of Weight Stigma and Weight History on Self

This super-ordinate theme comprised of three sub-ordinate themes, which are important when looked at in relation to one another. They do not appear to be separate entities, but interact together, leading to the development of restrictive eating behaviours. The sub-ordinate themes consisted of “Influence of past life experiences related to weight on the present”, “Fear of putting on weight and going back to before”, and “Struggling with my mind—internal battles”.

All participants spoke about how past life experiences influenced their current experience of life post-bariatric surgery. All of the women describe long-standing weight issues, with most describing themselves as being overweight from a young age. Memories regarding weight played on their mind and negatively interacted with how they viewed themselves. The longevity of the weight issues seemed to be pivotal in the current restrictive behaviours. The women had struggled with their weight for such a long time it may be that the associated negative memories (e.g. bullying) and negative reactions from other people (both strangers and people they knew) regarding their weight before surgery had become particularly salient because so much emotion and distress was associated with them. “I can vividly remember how I felt before and what people used to say to me and walking down the street and what people would shout out to you I can physically still remember how that made me feel” (Participant 4). These memories, which were often intrusive, led the women to develop intense fears around regaining the weight they had lost from surgery. They became hyper-vigilant and anxious about their weight, as they wanted to avoid going back to how they were before the surgery, leading to the restrictive eating behaviours. “I don’t enjoy eating I really don’t enjoy eating now I hate eating I just don’t like eating cos it’s constantly on my mind when I put something in my mouth I’m gonna get fat” (Participant 2). All of the women spoke about previous experiences of weight gain and attempts at weight loss, which had all been unsuccessful. Participants reported that it was this yo-yo dieting and continuous disappointment with non-surgical weight loss interventions that had led to them seeking bariatric surgery.

The past experiences of the women and their fear of weight gain contributed to the negative way in which they thought about themselves “… anything to do with my weight, body image, food all fall into the ‘this is why you are crap’ category without fail” (Participant 1). The surgery was viewed as a way to move away from these experiences; however, there was a sense that the weight stigma experienced by these women had been internalised and contributed to the restrictive eating behaviours. “I don’t think I’ve quite accepted the fact that I’ve done it I’ve lost weight I think I’m still punishing myself for being that girl that ate all that food” (Participant 5).

### The Impact of Loose Skin

This super-ordinate theme comprises of three sub-ordinate themes entitled “It reminds me of what I was before—excess skin as a reminder”, “You can’t tell I’ve lost weight because of it—excess skin hides weight loss” and “I look like melted candle woman—excess skin is unsightly”. Each sub-ordinate theme related to a different negative aspect of how the presence of excess skin impacted on the participants. They also mapped on to the themes of the past, weight gain and views about the self that were present in the previous super-ordinate theme above, adding additional “fuel” to the desire to carry out restrictive eating disordered behaviours.

The participants’ accounts were interspersed with descriptions of how the presence of excess skin masked their weight loss from the bariatric surgery. The main reason for this seemed to be the different way the skin “hung” on the body. The skin folds created a silhouette that was different to the smooth outlines expected. The women often described not being able to tell they had lost weight due to the excess skin disguising weight loss and giving a false image of their true weight. “My excess skin that’s the main reason why I won’t look in the mirror the reason why I still don’t feel there’s any change because of the amount of excess skin I’ve got it still makes me look fat and it’s still there to remind me of my fat and it’s just horrible” (Participant 2).

A significant negative impact of the excess skin was its appearance, with the texture of “empty” skin being different to when it was “full”. “What no one was calculating till now is actually the impact of what it’s like to look in the mirror […] I look like melted candle woman, or a balloon that’s been popped and it’s all wrinkly and stuff with all the excess skin” (Participant 1). The women spoke of their disgust and hatred towards the excess skin and the negative impact it had on their self-confidence. The appearance of excess skin interacted with participants’ negative cognitions and restrictive eating behaviours by acting as a reminder of past eating and the women’s pre-surgery weight. The women were aware that restricting would not improve the excess skin, but its presence led to the women fearing they were more susceptible to regaining weight. “I feel if my excess skin weren’t there I think I’d be a lot more (pause) I dunno coping with the weight loss recognising it like noticing it and I’d be a lot more confident because the excess skin’s there alright it may not be fat no more but it still looks like fat and it’s still there a constant everyday reminder and that’s the most thing I hate about my body it’s ergh it’s there and it’s just rank” (Participant 2).

### Thoughts About Food and Disordered Eating Patterns

The women’s thoughts about food changed following surgery. They described food in a more functional manner, which led to less being consumed. “For me it’s very much a fuel it’s not an enjoyable thing” (Participant 1). In addition, there was a sense that food was a threat—“unsafe”, “the enemy”—and had to be avoided. “I don’t want food to be my enemy again and do that to me again so that’s why I try to stay away from you know certain foods” (Participant 3). There seemed to be a pattern of guilt around eating and not deserving food—food was not to be enjoyed and was viewed as a punishment. “On the few moments where I’m like oh this tastes really nice it’s really nice to be out with my friends and this is good fun and then the guilt comes in like a massive pile of bricks and then I’ll just go really quiet and I’ll just hate it hate myself” (Participant 1). The women described “eating with their eyes”, so even though the women had not eaten the food (and rationally understood this) because they had seen it they felt like they had eaten it, leading to food avoidance.

Disordered eating behaviours included calorie counting, restriction, self-induced vomiting and the use of laxative-like medicine. They were carried out with the aim of “getting rid” of food that had been eaten and limiting the amount of calories absorbed so that weight was not impacted. The “drive” for carrying out these behaviours was exacerbated by the themes described above including memories of the past and fears about going back to their previous lives and difficulties, negative thoughts about the self and the presence of excess skin. The fears led the women to become overly strict, possibly not trusting that their body would not naturally regain the weight (as it had in previous weight loss attempts) unless they were exceptionally strict.

## Discussion

This study aimed to explore the lived experiences of women who had developed restrictive eating behaviours after bariatric surgery. Analysis revealed three super-ordinate themes “Experiences of weight stigma and weight history on self”, “The impact of loose skin” and “Thoughts about food and disordered eating patterns”.

All the women referenced how past weight-related experiences played on their mind and led to an increased fear of regaining their lost weight and going back to life pre-surgery. This stemmed from negative stigmatising experiences from others, in addition to their own internalised self-directed stigma regarding living with obesity. Research has shown that individuals seeking bariatric surgery describe a negative pre-surgery quality of life stemming from weight related stigmatization, and adverse experiences have been implicated in the development of AN, with teasing, criticism and bullying associated with increased risk [2, 30, 46–49].

It is hypothesised that internalised weight bias—where people internalise other people’s weight stigma, society’s anti-fat attitudes and negative weight stereotypes—may have led to restrictive eating behaviours in an attempt to avoid regaining lost weight [24, 50–53]. The higher a person’s internalised weight bias, the greater their body image concerns, depression, anxiety, stress and low self-esteem. Higher levels of internalised weight bias relate to increased eating disturbance and higher drive for thinness which have been associated with restrictive eating disorders, such as AN [51, 52, 54–57]. Assessment may benefit from measuring internalised weight bias as well as past experiences of weight-related stigma [21, 47]. Further research could focus on whether past negative experiences, internalised weight bias and body image concerns are more apparent in people with post-surgery restrictive eating disordered behaviours compared to general post-surgery patients and whether training in resilience, empowerment and self-compassion can combat the effects of internalised weight bias [58, 59].

Excess skin may have played a significant role in the development of restrictive eating behaviours by acting as a reminder of the past, weight stigma and the associated distress. The women described their loose skin as unsightly, leading to feelings of shame and embarrassment, all of which have been shown to impact negatively on body image [33, 34, 60, 61]. Although the women knew the restrictive behaviours would not improve the excess skin, its presence was enough to incite fear around weight gain and perpetuate the disordered eating. Women may be more impacted by excess skin due to the areas typically affected being associated with feminine beauty (breasts, upper arms, thighs) [33].

All the women in this study would have liked body contouring surgery (BCS) and desire following bariatric surgery can be as high as 88%, with one of the main drivers being the presence of excess skin [62–64]. However, BCS is considered cosmetic, rather than reconstructive or functional surgery, and is separate from the bariatric pathway [65]. BCS is an invasive and costly intervention and although it can improve quality of life and reduce body image concern, scores are often still above non-clinical levels [22, 30, 31, 62, 66]. Monitoring discontent and preoccupation with excess skin, its impact on body image and association with dietary restriction may identify those at risk of developing restrictive eating behaviours after bariatric surgery [67, 68]. As part of their ongoing care following bariatric surgery, it may be beneficial for services to provide psychological support and body image interventions, even if people have BCS [22, 67, 69]. There are a range of interventions that could benefit individuals (especially if excess skin is present) targeting specific concerns, from exploring feelings of shame and body image acceptance, to reducing body distortion [24, 29, 70]. Further research should examine whether outcomes are improved in those who have BCS, compared to those who do not, and whether BCS should be integrated into the bariatric surgery pathway [65, 71].

The theme “Thoughts about food and disordered eating patterns” highlighted changes to how food was perceived. The majority described food as being a necessity rather than a source of enjoyment, which maintained the restrictive eating. Malnutrition can lead to changes in the brain which impact on neurocognitive processing leading to increased rigidity [72, 73]. The women’s rapid weight loss may have maintained maladaptive cognitions and behaviours through increased preoccupation/obsessionality with details of diet/shape/weight and increased weight and eating control behaviours [55, 74]. Rigid habits may have developed as the women reported preoccupation with food and following rules around eating, such as calories, what food was “safe” or to be avoided, as well as cooking methods. Breaking these rules led to distress and guilt and intensified fears around weight gain which perpetuated unhelpful beliefs and behaviours. There could be a role for training around how professionals may unknowingly reinforce unhelpful behaviours, for example by congratulating a patient on losing weight. Changing the language professionals use could reduce the effects of weight-related stigma, such as using terms weight, BMI and heaviness rather than fatness, obesity and excess fat [75, 76].

One of the difficulties in identifying patients with restrictive eating disordered behaviours following bariatric surgery is that they are often seen as success stories due to the amount of weight lost. Weight loss is understandably an important variable; however, the impact of this unique dramatic weight loss on the psychological well-being of people who were once extremely obese still remains to be fully understood [29, 77, 78]. There is a growing consensus that definitions of successful outcomes after bariatric surgery should not solely focus on weight loss and improvement of co-morbid medical conditions but also on improved quality of life and psychosocial functioning such as social and personal adjustment, positive changes on pre-operative psychological parameters, satisfaction with outcome and confidence in ability to adopt or maintain new behaviour patterns [18, 24, 26, 67].

Linked with this is a debate around what is unusual/expected weight loss following bariatric surgery [26]. To have a BMI of 25 kg/m^2^ following surgery is extremely unusual and likely to have been achieved through highly restrictive eating. The women reported behaviours and cognitions that map on to the DSM-V criterion for AN, but due to their weight met the criteria for Other Specified Feeding or Eating Disorder (OSFED)—Atypical AN [13, 14]. This calls in to question whether setting “ideal” BMI at 25 kg/m^2^ [16], even though this is considered normal in the general population, is appropriate and may increase vulnerability to restrictive eating disorders.

These presentations are rare and under-reported in the bariatric surgery population [7], and the clinical significance of these behaviours can become lost. Future research would make a valuable contribution by exploring other indicators of a restrictive eating disorder, such as assessing laboratory evidence and physical manifestations of malnutrition. The study inclusion criteria ensured homogeneity [39] but highlighted areas for future investigation, such as exploration of the male experience. In this study, the time elapsed post-surgery ranged from nine months to eight years, creating challenges when concluding if the findings reflect the shared lived experience. However, all the women had the same surgical procedure of a Roux-en-Y gastric bypass, which strengthens confidence in the findings and the likelihood that the themes reflect the experience of the broader population. Roux-en-Y gastric bypass results in a quicker rate of weight loss than other commonly performed types of surgery, and people receive more physiological feedback after eating which could intensify eating disorder cognitions. Future research could explore if this presentation is seen in individuals who have had other types of bariatric surgery.

## Conclusions

Bariatric surgery is an effective treatment for severe obesity when other interventions have been unsuccessful. Understanding of post-surgery eating pathology is increasing, but the overall body of literature is still limited and the prevalence of post-surgical excessive dietary restriction remains unknown. This is one of the first qualitative studies with people who have developed restrictive eating disordered behaviours following bariatric surgery and highlights the complexities involved in the development of these behaviours. These interviews highlight the important role of weight stigma, and this would be a useful route to be explored in future research. It is hoped that this study may help clinicians distinguish AN eating pathology from necessary post-surgery dietary adjustment, so that people suffering from these conditions are provided with an appropriate framework to identify and legitimise their symptoms. This study demonstrates that expanding our understanding of this presentation is critical in order to support early identification and improve people’s lives and mental well-being following bariatric surgery.
